# Octogenarians and Pancreatic Cancer: Is Surgery Always the Right Choice?

**DOI:** 10.3390/cancers18152423

**Published:** 2026-07-28

**Authors:** Lennart von Fritsch, Dina Abual Naaj, Jannis Duhn, Kim C. Honselmann, Louisa Bolm, Rüdiger Braun, Hryhoriy Lapshyn, Stanislav Litkevych, Constanze Schneider, Fabian Reinwald, Andrea Sackmann, Monika Klinkhammer-Schalke, Sylke Ruth Zeissig, Bianca Franke, Richard Hummel, Kerstin Weitmann, Simone Faißt, Ian Wittenberg, Jessica Isabel Selig, Soo-Zin Kim-Wanner, Wolfgang Hoffmann, Tobias Keck, Ulrich F. Wellner, Steffen Deichmann, Thaer S. A. Abdalla

**Affiliations:** 1 Department of Surgery, University of Lübeck, 23562 Lübeck, Germany; 2 Clinical-Epidemiological Cancer Registry Brandenburg-Berlin, 03044 Cottbus, Germany; 3 Cancer Registry of Rhineland-Palatinate, Institute for Digital Health Data RLP, 55101 Mainz, Germany; 4 Hessian Cancer Registry, Hessian Office for Health and Care, 60439 Frankfurt am Main, Germany; 5 Association of German Tumor Centers (ADT) (Arbeitsgemeinschaft Deutscher Tumorzentren e.V.), 14057 Berlin, Germany; 6 Institute of Clinical Epidemiology and Biometry (ICE-B), University of Würzburg, 97070 Würzburg, Germany; 7 Department of Surgery, University Medical Center Greifswald, 17475 Greifswald, Germany; 8 Central Unit of the Cancer Registry Mecklenburg-Western Pomerania, 17475 Greifswald, Germany; 9 Institute for Health Services Research at the Oncology Center Stuttgart e.V., 70176 Stuttgart, Germany; 10 Cancer Registry Saxony-Anhalt, 06112 Halle (Saale), Germany; 11 Cancer Registry Saxony, 01099 Dresden, Germany

**Keywords:** PDAC, pancreatic cancer, neoadjuvant treatment, octogenarians

## Abstract

**Background:** Demographic change is leading to an increasing portion of octogenarians with pancreatic ductal adenocarcinoma (PDAC). Surgery remains a central component of curative-intent treatment. Nevertheless, it carries a risk of potentially life-threatening complications and high morbidity. Whether octogenarians benefit from surgery remains unclear. **Methods:** Using data from the clinical cancer registries of the 10th National Oncology Quality Conference of the ADT, we divided all patients with PDAC diagnosed between 2015 and 2023 into three age groups (≤65 years, *n* = 944; 66–79 years, *n* = 1535; ≥80 years, *n* = 537) and compared demographics, histopathology, treatment patterns, and survival data. **Results:** We observed an increasing proportion of octogenarians over the years from 15% in 2015 to 32% in 2023. While they were diagnosed with more favorable tumor stages, they more often received palliative chemotherapy (28% vs. 18%/19% in the younger cohorts). Only 24% of octogenarians who underwent surgery received adjuvant chemotherapy. Octogenarians had a worse overall and disease-free survival and 90-day mortality than the younger age groups. Furthermore, octogenarians with surgery or chemotherapy with curative intent alone had survival rates comparable to patients with palliative care. **Conclusions:** Octogenarians largely do not benefit from surgery alone. As only a quarter of octogenarians received adjuvant chemotherapy after surgery, neoadjuvant concepts might be useful for selecting patients who are fit enough to undergo and benefit from multimodal treatment.

## Simple Summary

This study looked at older patients aged eighty years or more with pancreatic cancer. The number of these patients increased between 2015 and 2023. Although their cancer was often found at an earlier stage, their outcomes were worse than in younger patients. Surgery can be an important treatment for pancreatic cancer, but it also has high risks. In this study, older patients had higher death rates after treatment. Many older patients who had surgery did not receive chemotherapy afterwards. Patients who had only surgery or only chemotherapy with the aim to cure had similar survival to patients who received treatment only to relieve symptoms. The results suggest that surgery alone is often not enough for very old patients. Giving chemotherapy before surgery may help doctors find patients who are fit enough to benefit from stronger combined treatment.

## 1. Introduction

Demographic change places substantial strain on health care systems in developed countries. In addition to rising costs and shrinking workforces, health care providers are increasingly confronted with patients aged 80–89 years (octogenarians) seeking treatment for complex conditions. Simultaneously, cancer has emerged as an escalating global burden, surpassing cardiovascular diseases as the leading cause of death in many regions [1]. Pancreatic cancer is among the most commonly diagnosed malignancies in Germany [2]. Despite significant advances in diagnosis and treatment, the most common subtype—pancreatic ductal adenocarcinoma (PDAC)—continues to carry a dismal prognosis, with 90% of patients surviving less than five years [3]. Embedded in a multimodal treatment strategy, complete surgical resection remains a central part of curative-intent treatment, despite being a technically demanding and radical procedure. Pancreatic surgery is associated with potentially life-threatening complications, such as postoperative pancreatic fistula, which occurs in 10–14% of patients [4,5,6,7,8], and post-pancreatectomy hemorrhage, which occurs in 2.9–27.7% of cases [9,10], resulting in an in-hospital mortality rate of 2.8–6.5% [11,12,13,14].

Given the potential severity of postoperative morbidity, careful patient selection remains essential, as not all patients are suitable candidates for surgery. However, excluding a patient from surgical treatment markedly reduces the expected survival, making this decision one that must be carefully weighed in a multidisciplinary patient-centered discussion.

A selection criterion frequently discussed is age. Advanced age is inherently associated with increased frailty and comorbidity. Reduced muscle mass and physical strength, along with a higher prevalence of chronic conditions, may predispose one to higher complication rates. Whether these risks outweigh the potential survival benefit of surgical intervention compared with palliative therapy remains uncertain. Nevertheless, given a remaining life expectancy of an 80-year-old in Germany of 8 years for men and 9.6 years for women [15], many patients might still benefit from curative-intent treatment.

To date, few studies have examined outcomes according to completion of the intended treatment strategy in this vulnerable population. Using a large German registry-based cohort, we therefore analyzed the entire treatment pathway in octogenarians with non-metastatic PDAC and assessed whether curative-intent monomodal treatment confers a survival advantage over palliative management. Furthermore, we compared the treatment patterns, perioperative outcomes, and survival with those of younger patients.

## 2. Materials and Methods

### 2.1. Patient Cohort

The study was carried out according to the regulations of the German Cancer Registry Group (GCRG). Ethical approval was obtained from the ethics committee of the University of Lübeck. As part of the project for the 10th National Conference on Quality in Oncology, held in conjunction with the 2024 German Cancer Congress, data from the state cancer registries and facility-based registries were requested and compiled by the Association of German Tumor Centers (ADT). The data of patients with an invasive PDAC (morphology 8500/3 or 8140/3) of the pancreas diagnosed between 2015 and 2023, including information on Eastern Cooperative Oncology Group Performance Status (ECOG) at diagnosis and treatment, were selected. Patients with distant metastases at diagnosis (M1) were excluded.

As the TNM-classification had been updated between 2015 and 2023, cN status was dichotomized into cN0 or cN+. To stratify by age, the cohort was split into 3 subgroups (≤65 years, 66–79 years, ≥80 years).

To assess the potential selection bias arising from the exclusion of patients with missing data, we compared the baseline characteristics and overall survival in the cohorts before and after exclusion.

### 2.2. Statistical Analyses

Calculations were performed using R software (R Core Team (2024). R: A Language and Environment for Statistical Computing. R Foundation for Statistical Computing, Vienna, Austria, V4.4.1). Statistical significance was set to *p* < 0.05. Cases with missing data were excluded per analysis. Continuous variables were reported as medians and interquartile ranges (IQR) and nominal variables using frequencies. Group differences were assessed using the Kruskal–Wallis test for continuous variables and the chi-square test for categorical variables. Overall survival (OS) and disease-free survival (DFS) were defined as the time from the date of diagnosis/procedure to the date of death/recurrence or end of follow-up. Survival analyses were performed using the Kaplan–Meier method and the log-rank test.

#### 2.2.1. Assessment of Whole Cohort

To explore the influence of age on the outcome of surgery, we performed multivariable logistic regression with age, sex, pT, pN, ECOG performance status, receipt of neoadjuvant treatment, and type of procedure (pancreatic head resection [PD], distal pancreatectomy [DPR] or total pancreatectomy [TPE]) as predictors and 90-day mortality as the endpoint.

#### 2.2.2. Assessment of Octogenarians

The treatment flow of octogenarians was visualized using the PantaRhei-package, V0.1.2 [16].

To assess the treatment effect in octogenarians, we split the cohort into patients receiving palliative treatment, a multimodal treatment (i.e., surgery combined with (neo-adjuvant chemotherapy) and non-multimodal/monomodal treatment (neoadjuvant chemotherapy without surgery or surgery alone). The difference between neoadjuvant chemotherapy alone and palliative treatment was based on the initial treatment intention (curative/palliative). We performed Kaplan–Meier analysis for OS. To address potential immortal time bias, patients who died within the first 90 days after surgery were excluded from this analysis. Furthermore, the analysis was repeated as a sensitivity analysis after excluding cases treated with neoadjuvant therapy.

To adjust for potential confounders and further reduce the risk of immortal time bias, we conducted a time-dependent Cox regression, including sex, age, ECOG performance status, and nodal status as independent variables. The treatment approach was modeled as a time-dependent categorical covariate. The time from diagnosis was divided into start–stop intervals, with the treatment status updated when a new treatment modality was received. Thus, unlike in the preceding Kaplan–Meier analysis, patients contributed survival time to different treatment categories as their treatment status changed over time.

Furthermore, we performed a binary regression with receipt of multimodal treatment as the endpoint using age, sex, cT-status, cN-status, ECOG performance status, and tumor location as input variables.

This study was conducted in reference to the STROBE guidelines for cohort studies [17].

## 3. Results

### 3.1. Descriptive Statistics

**Figure 1:**
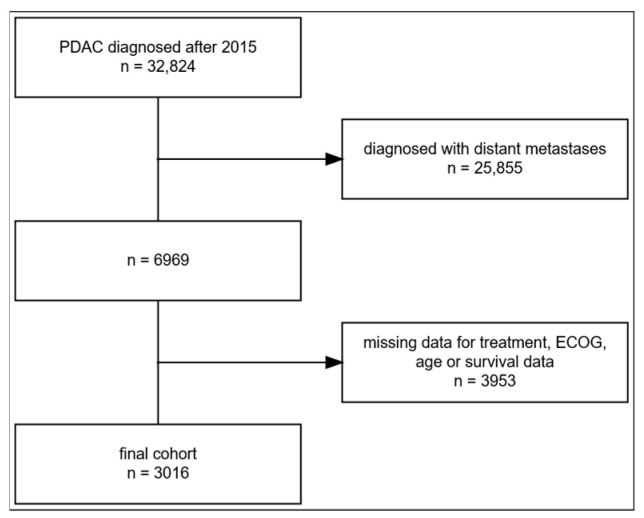
Inclusion flowchart of patients in the participating registries diagnosed with pancreatic adenocarcinoma (PDAC) after 2015. ECOG = Eastern Cooperative Oncology Group Performance Status.

**Figure 2:**
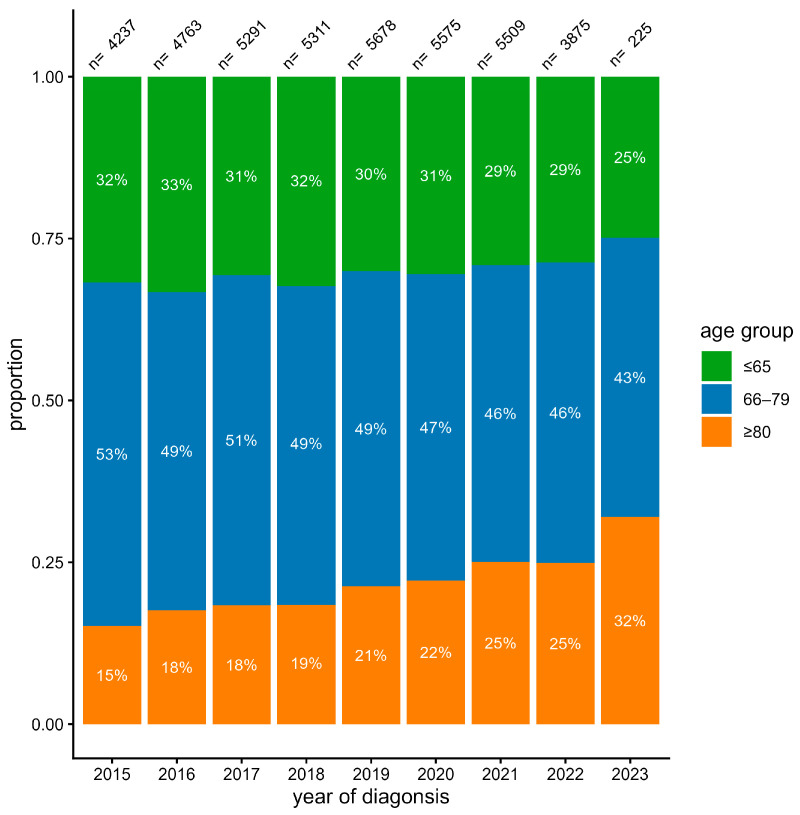
Share of different age groups of all patients diagnosed with PDAC over the years in Germany. Number in 2023 reduced to delayed data delivery of registries.

Among 32,824 patients diagnosed with pancreatic cancer between 2015 and 2023 in the participating registries, 25,855 presented with distant metastases at initial diagnosis. After excluding patients with missing data on age, treatment, ECOG performance status, or survival time, 3016 patients remained in the final cohort (Figure 1). To assess the potential selection bias resulting from these exclusions, baseline demographic and histopathological characteristics, as well as overall survival, were compared before and after exclusion. No significant differences were observed, except for a decrease in the proportion of patients undergoing upfront surgery from 67.1% to 63.6% after exclusion (*p* = 0.007; Supplementary Figure S1 and Supplementary Table S1). Data completeness in the final cohort was high, with 78% of variables having less than 10% missingness (Supplementary Figure S2). The median age was 71 years (IQR 63–78), and 49.3% were female. The group aged ≤65 years included 944 patients (31.3%), with a median age of 59 years (IQR 55–63), and 46.0% were female; the group aged 66–79 years (*n* = 1535, 50.9%) had a median age of 73 years (IQR 69–76) with 49.3% females; and the group aged ≥80 years (*n* = 537, 17.8%) had a median age of 82 years (IQR 81–84) with 55.1% females (Table 1). We observed a steady annual increase in the proportion of octogenarians among all patients, raising from 15% in 2015 to 32% in 2023 (Figure 2).

**Figure 3:**
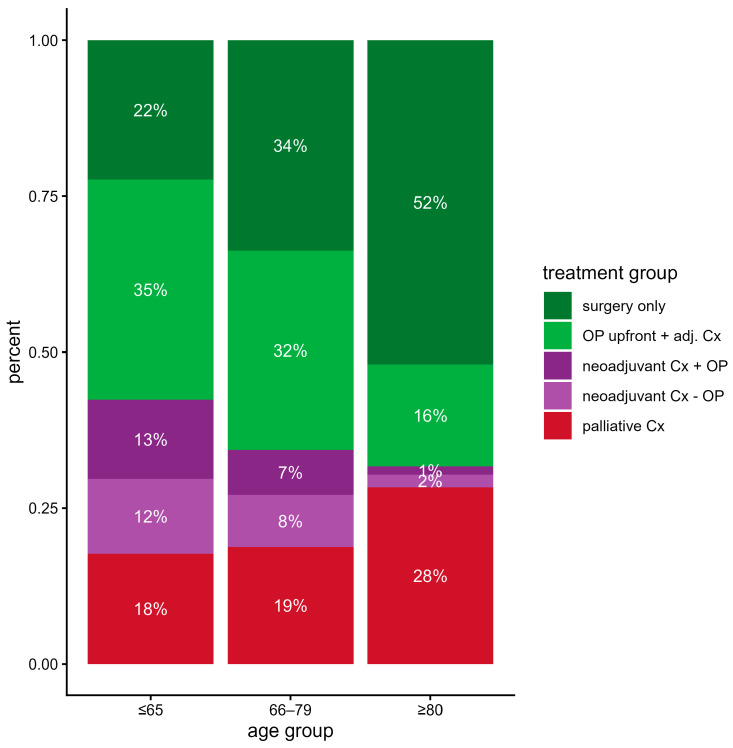
Share of treatment modalities in percent for PDAC without metastases at diagnosis in different age groups. Cx = chemotherapy, OP = surgery. The difference between neoadjuvant chemotherapy without surgery (“-OP”) and palliative treatment was based on the initial treatment intention (curative or palliative) at the start of treatment.

**Figure 4:**
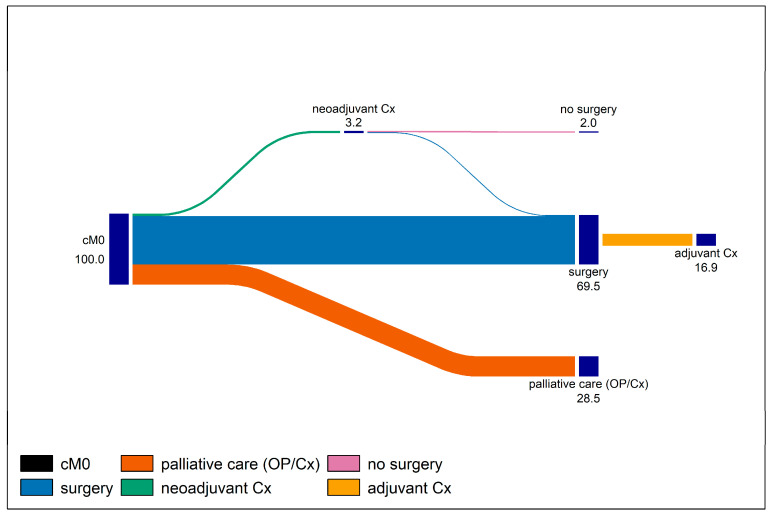
Treatment path in octogenarians with no clinical evidence of metastases (cM0). Percentages as share of total cohort.

Regarding clinical tumor characteristics, increasing age was associated with smaller tumors as reflected by the cT-status and less lymph node involvement (Table 1). Octogenarians more often received upfront surgery or palliative chemotherapy; whereas only 18% of patients aged ≤65 years received palliative treatment, 28% of octogenarians did (*p* < 0.001) (Figure 3). Only 3.4% of octogenarians underwent neoadjuvant treatment, and almost 60% of these patients did not advance to surgery. Of those octogenarians undergoing surgery, 24.3% received adjuvant treatment, compared to 57.5% for ≤65-year-olds and 47.6% for 66–79-year-olds (*p* < 0.001) (Table 1/Figure 4).

### 3.2. Survival Rates

**Figure 5:**
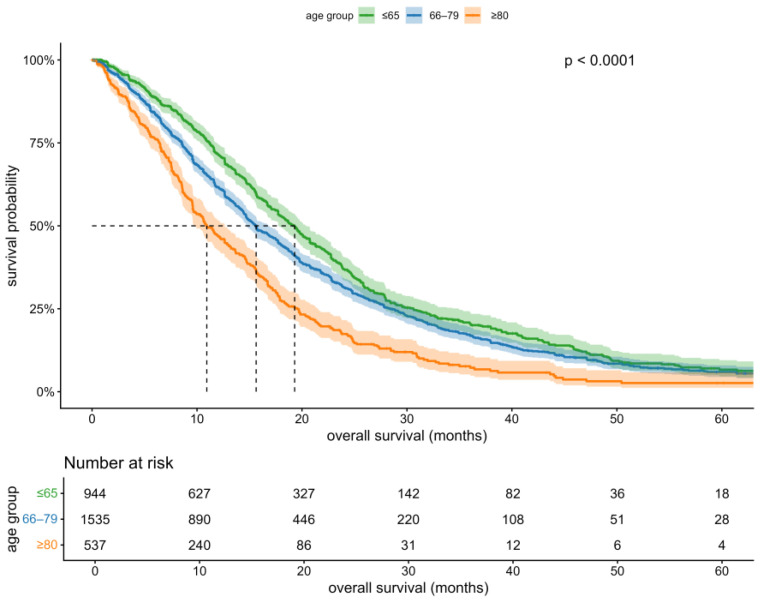
Overall survival (OS) of PDAC patients in different age groups without metastasis at diagnosis. Median OS (dashed lines) ≤ 65 years (green) = 19.3 months, median OS 66–79 years (blue) = 15.6 months, median OS ≥ 80 years (orange) = 10.9 months, log-rank test *p* < 0.0001.

The median OS was 19.3 months (95% confidence interval [95%CI] 17.8–20.4) for patients aged ≤65 years, 15.6 months (95%CI 14.7–16.9) for those aged 66–79 years, and 10.9 months (95%CI 9.6–12.6) for those aged ≥80 years (log-rank *p* < 0.001) (Figure 5).

### 3.3. The 90-Day Mortality Rate After Surgery

The 90-day mortality after PD was 5.3% for patients aged ≤65 years, 8.6% for patients aged 66–79 years, and 16.9% for patients aged ≥80 years (*p* < 0.001). After DPR, the 90-day mortality was 5.8% for patients aged ≤65 years, 4.0% for patients aged 66–79 years, and 12.2% for patients aged ≥80 years (*p* = 0.11). After TPE, the 90-day mortality was 14.7% for patients aged ≤65 years, 21.9% for patients aged 66–79 years, and 27.3% for patients aged ≥80 years (*p* = 0.27) (Supplementary Figure S3a–c).

### 3.4. Multivariable Logistic Regression for 90-Day Mortality

To further assess the influence of age on patients undergoing surgery, we performed a multivariable logistic regression analysis. In this model, age ≥ 80 years was a strong predictor of 90-day mortality with an odds ratio (OR) of 3.00 (95%CI 1.89–4.83, *p* < 0.001) compared to patients ≤ 65 years. Furthermore, poorer ECOG performance status (2–4) was associated with higher 90-day mortality compared to ECOG 0-1 (OR 2.26 [95%CI 1.47–3.42], *p* < 0.001). Among the types of surgery, TPE carried a significantly higher 90-day mortality risk compared to PD (OR 2.82 [95%CI 1.92–4.09], *p* < 0.001), while DPR did not (*p* = 0.213). Female sex was associated with decreased 90-day mortality compared to male sex (OR 0.61 [95%CI 0.44–0.84], *p* = 0.002) (Supplementary Figure S4).

### 3.5. Disease-Free Survival After Surgery

DFS data were available for 1618 patients who underwent surgery. DFS differed significantly between the age groups (*p* < 0.001). The median DFS was 13.7 months (95%CI 12.9–15.3) for those ≤65 years and 14.5 months (95%CI 13.6–15.4) for those 66–79 years. Patients aged ≥80 years had a markedly shorter DFS, with a median of 10.4 months (95%CI 9.3–13.2, *p* < 0.001) (Supplementary Figure S5).

### 3.6. Demographics of Octogenarians by Treatment Modality

For further analyses, we grouped all octogenarians into three cohorts (multimodal, non-multimodal and palliative), as described in the Methods section. Demographics can be found in Table 2. Patients receiving palliative treatment had a more advanced tumor stage (*p* < 0.001), and the tumor was more often localized in the corpus or tail (*p* = 0.004).

### 3.7. Overall Survival by Treatment Modality

**Figure 6:**
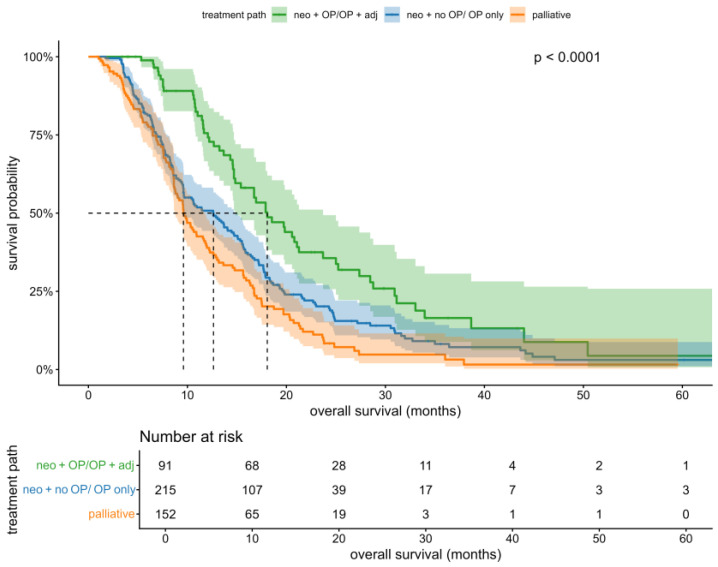
Overall survival (OS) by treatment modality in octogenarians with PDAC. Median OS (dashed lines) multimodal treatment (green) = 18.1 months, median OS monomodal (blue) = 12.6 months, median OS palliative (orange) = 9.6 months, log-rank test *p* < 0.0001, neo = neoadjuvant treatment, adj = adjuvant treatment, OP = surgery.

Octogenarians receiving non-multimodal treatment had a median OS of 12.6 months (95%CI 9.6–15.1) and were therefore comparable to those with palliative treatment (median OS 9.6 months [95%CI 8.8–11.8]). Octogenarians receiving multimodal treatment had a median OS of 18.1 months (95%CI 14.8–23.7) (*p* < 0.001) (Figure 6). Analyses repeated after excluding cases treated with neoadjuvant therapy produced consistent results. In the younger cohorts, however, patients benefited more from monomodal over palliative treatment (Supplementary Figure S6). To control for potentially confounding factors, we performed a time-dependent Cox regression analysis including only octogenarians. In the resulting model, multimodal treatment (hazard ratio [HR] 0.63 [95%CI 0.45–0.90], *p* = 0.01) had a positive impact on survival compared to palliative care, whereas monomodal treatment did not (HR 1.07 [95%CI 0.83–1.36], *p* = 0.61). Besides this, lymph node involvement, older age, and poorer ECOG performance status were associated with worse OS (Supplementary Figure S7). To identify patients who might benefit from surgery alone, we stratified octogenarians undergoing surgery without (neo-)adjuvant treatment by the third quartile of OS (15.7 months) and compared tumor histopathology, location, and the surgical procedure performed. Patients with a longer OS had a more favorable histopathology in terms of tumor size (cT1/2: 84.5% vs. 60.9%, *p* = 0.08; pT1/2: 64.7% vs. 54.1%, *p* = 0.03; upper 25% vs. remaining patients, respectively) and rates of lymph node involvement (cN0: 79.5% vs. 56.7%, *p* = 0.01; pN0: 50.0% vs. 32.5%, *p* = 0.02; upper 25% vs. remaining patients, respectively). No differences were observed in ECOG performance status, sex, tumor location, or the type of surgery performed.

### 3.8. Assessing the Chance of Receiving Multimodal Treatment in Octogenarians

As we observed a survival benefit for octogenarians receiving multimodal treatment, we performed a binary regression analysis to identify preoperative factors associated with receiving multimodal treatment. Patients treated with palliative intent were excluded. Age (always above 80, as only octogenarians were included in this analysis) was included as a continuous variable. In the resulting model, no independent variable besides age (*p* = 0.005) was associated with the endpoint (Supplementary Figure S8).

## 4. Discussion

In this study, we assessed the oncological outcomes of octogenarians with non-metastatic PDAC compared to younger patients. Despite having more favorable T and N status, octogenarians had worse OS and a higher 90-day mortality irrespective of the type of procedure, primary tumor features and baseline performance status. Furthermore, only octogenarians receiving multimodal therapy achieved longer survival times compared to monomodal treatment. When failing to receive multimodal treatment, the OS was comparable to that of patients receiving palliative treatment.

As we know, Europe’s population is rapidly aging. As a result, we are expected to encounter more octogenarians with PDAC in our daily practice. Our study shows an incremental increase in the proportion of octogenarians, doubling from 15% in 2015 to 32% in 2023. This aligns with data from the Robert Koch Institute, which show that the incidence rate in Germany increased from 104.4 per 100,000 inhabitants among 80–84-year-olds in 2015 to 112.6 in 2023 [18]. As personalized treatment strategies in current international guidelines are lacking [19,20,21], this should be considered an important unmet need.

Other studies have likewise reported reduced OS among octogenarians [22,23]. This is not a surprising finding, as life expectancy is influenced by age itself. Frailty and higher numbers of comorbidities are common with advancing age and can increase the risk of complications in the context of invasive procedures. Therefore, the higher risk of mortality needs to be carefully balanced against the intendent benefits of treatment. In our study, we observed a higher 90-day perioperative mortality across all types of surgery (PD: 16.9%, DPR: 12.2%, TPE: 27.3%) for octogenarians compared to younger cohorts. Similarly, Vigneron et al. found a markedly higher 90-day mortality of 15.4% for octogenarians compared to 70–79-year-olds (5.3%) and <70-year-olds (2.9%) after PD [24]. Matsui et al. found an in-hospital mortality rate of 6.9% for patients > 80 after radical resection [23]. In a review by Riall, mortality after pancreatic resection was increased across 10 institutional and two population-based analyses for older cohorts [25]. We performed a multivariable logistic regression for 90-day mortality and found age to be the strongest predictor, followed by ECOG, procedure type, and sex. Likewise, Hackner et al. found a higher in-hospital mortality for older patients and, furthermore, found age to be an independent prognostic factor for OS [22].

In a 2018 study based on the Surveillance, Epidemiology, and End Results database, a marked influence of age on the treatment course was observed. Among 5975 pancreatic cancer cases, 34.6% of patients > 75 received no treatment versus 9.1–12.2% in younger cohorts; logistic regression confirmed that age was associated with the likelihood of receiving any treatment [26]. Despite milder tumor characteristics at diagnosis, 28% of octogenarians received palliative chemotherapy compared with 18–19% in younger groups in our study. Although causality cannot be inferred from our data, this may be attributable to poorer ECOG performance status in octogenarians (16.6% ECOG 2–4 vs. 5.3% in ≤65-year-olds).

In our study, octogenarians had a median postoperative DFS of 10.4 months, which was significantly shorter compared to both younger cohorts (13.7 months for ≤65 years, 14.5 months for 66–79) and was consistent with findings from other studies [22,27]. Hackner et al. also found that advanced age (>70 years) was associated with shorter DFS, with a median DFS of 10.4 months for the older cohort after pancreatic resection [22]. Our data suggest that failure to receive multimodal treatment may contribute to this finding, consistent with a plethora of studies demonstrating the benefits of adjuvant therapy [23,27,28,29,30,31]. Matsui et al. assessed the rate of adjuvant chemotherapy in four different age groups after pancreatic surgery and found that octogenarians had the highest rates of not receiving any adjuvant treatment (56.9%) and discontinuing treatment (59.1%) [23]. In our cohort, only 24% of octogenarians who underwent surgery eventually received adjuvant chemotherapy compared to 58% in patients ≤ 65 years and 48% in patients aged 66–79 years. Similarly, only 41% of octogenarians with neoadjuvant treatment eventually underwent resection, which is dismal compared to the 90% reported in the NORPACT-1 trial [32]. The reasons for not undergoing surgery or receiving adjuvant chemotherapy are likely multifactorial. In a study by Mackay et al. involving 1306 patients who underwent pancreatic surgery, 33% did not receive adjuvant chemotherapy. Independent predictors included older age, poorer ECOG performance status, and postoperative complications, particularly pancreatic fistula and hemorrhage. In addition, the annual surgical case volume of the treating center was independently associated with the likelihood of receiving adjuvant chemotherapy [33]. Similar findings were reported by Poiraud et al. in a French nationwide study [34]. In our study, the reasons for omission of surgery or adjuvant chemotherapy could not be determined, because the registries do not capture data on postoperative complications, frailty, or quality of life. However, failing to receive multimodal treatment in octogenarians resulted in survival outcomes comparable to those observed with palliative treatment, a trend not observed in the younger cohorts. Likewise, Hue et al. observed a median survival difference of 3 months between chemotherapy alone and surgery alone, yet an almost doubled median survival for multimodal treatment [35]. In a cohort study by Mehtsun et al. of octogenarians following surgery, 47.4% received adjuvant chemotherapy, and this was associated with longer survival times [36]. A similar trend was noted by Sugawara et al.: for patients > 75 years, the median OS in the adjuvant treatment group was 19 months compared to 15 months for surgery only [37]. Despite this evidence, in a survey of 91 oncologists from 14 countries, 24% of the participants advised against adjuvant chemotherapy in octogenarians [38].

As multimodal treatment is crucial for improving OS compared with palliative approaches, we believe that a discussion of a revised treatment algorithm for octogenarians is warranted. In contrast to the low rate of neoadjuvant chemotherapy observed in our cohort, neoadjuvant treatment may represent a reasonable strategy for selected octogenarians, whereas upfront surgery could be considered primarily in patients with favorable biological and functional characteristics. Response to neoadjuvant therapy, together with preserved functional status, may help identify patients who are more likely to benefit from subsequent surgical resection. Conversely, patients with a poor treatment response or declining functional status could be spared a high-risk operation with limited expected benefit. These considerations, however, require confirmation in prospective studies.

The advantages and disadvantages of neoadjuvant chemotherapy remain a subject of ongoing debate. Neoadjuvant therapy permits early systemic drug delivery, which may reduce the burden of micrometastatic disease and increase R0 resection rates [39], a key determinant of favorable oncological outcomes [40]. Moreover, neoadjuvant treatment leverages tumor biology and time to identify patients most likely to benefit from surgical resection [41,42,43]. This effect may be particularly relevant in octogenarians. Critics of neoadjuvant approaches argue that initially resectable PDAC may progress to an unresectable stage during treatment. Accordingly, several studies have addressed or are currently addressing this issue. Some retrospective data suggest better OS with neoadjuvant therapy [44,45]. The NORPACT-1 trial, however, demonstrated no survival benefit with neoadjuvant treatment and suggested a trend toward an improved OS after upfront surgery [32]. In contrast, long-term results from the Dutch PREOPANC trial showed a superior 5-year OS in the neoadjuvant group (20.5% versus 6.5% in the intention-to-treat analysis) [46]. Likewise, the Japanese Phase II/III Prep-02/JSAP05 Trial showed a superior median OS of 37.0 months in the neoadjuvant group (gemcitabine plus S-1) compared to 26.6 months for upfront surgery [47]. Unfortunately, neither trial reported subgroup analyses of older patients.

In our study, octogenarians strongly benefited from a multimodal approach. However, in our cohort, no independent preoperative factor other than age predicted receipt of multimodal treatment. In a study from the nationwide Dutch Pancreatic Cancer Audit, age, performance status, annual caseload of the care delivering hospital and surgical complications were the key components influencing the probability of receiving adjuvant chemotherapy [33]. Similarly, in a French nationwide study, age over 80 years was associated with interruption or complete omission of adjuvant chemotherapy [34]. As such, the current staging and performance scoring systems are still inadequate to predict a patient’s fitness to complete a multimodal treatment. Thorough preoperative patient counseling regarding the need for adjuvant chemotherapy is warranted. In cases of refusal, the benefit of surgery should be carefully reconsidered. Additionally, the broad implementation of prehabilitation and routine frailty assessment should be discussed. The latter has been shown to independently correlate with OS in older patients and should therefore be considered when selecting a treatment modality [23].

We acknowledge the limitations of this study. It carries the established biases of retrospective studies. In particular, selection bias through missing data and underreporting cannot fully be ruled out. Furthermore, data on comorbidities, frailty, functional status, postoperative complications, quality of life, socioeconomic factors, and characteristics of the treating institutions were unavailable. These factors may substantially influence treatment selection and postoperative outcomes. Consequently, we could not determine whether the omission of surgery or adjuvant chemotherapy was related to perioperative morbidity, functional decline, or other unmeasured factors. The observed associations should therefore not be interpreted as evidence of causality. Despite these limitations, our study provides a comprehensive analysis of outcomes in octogenarians with pancreatic ductal adenocarcinoma on a national scale.

## 5. Conclusions

Octogenarians with pancreatic adenocarcinoma exhibit reduced OS and increased 90-day mortality across all surgical procedures compared with younger age groups. Moreover, only one quarter of octogenarians undergoing surgery received adjuvant therapy. Failure to receive multimodal treatment reduced the OS to levels comparable with palliative management. These findings underscore the importance of careful patient selection to identify octogenarians most likely to benefit from curative-intent surgery. The potential role of neoadjuvant and adjuvant treatment in this complex and vulnerable population should be further evaluated.

## Figures and Tables

**Table 1:** Descriptive statistics for the whole cohort and age subgroups.

	Whole Cohort	≤65 Years	66–79 Years	≥80 Years	
	(*n* = 3016, 100%)	(*n* = 944, 31.3%)	(*n* = 1535, 50.9%)	(*n* = 537, 17.8%)	*p*
**Age (years, median, IQR)**	71.0 (63.0–78.0)	59.0 (55.0–63.0)	73.0 (69.0–76.0)	82.0 (81.0–84.0)	<0.001
**Sex ratio (M:F)**	1:1	1:0.9	1:1	1:1.2	0.003
**cT (%)**					0.001
***Tis***	0.1	0.1	0.0	0.4	
***T*1**	7.7	7.0	7.4	9.7	
***T*2**	37.1	32.3	38.7	41.4	
***T*3**	28.5	29.0	29.2	25.7	
***T*4**	26.6	31.6	24.7	22.7	
cN * (%)					
***N*0**	52.7	48.5	52.6	60.5	<0.001
**ECOG † (%)**					
**0/1**	90.3	94.7	90.0	83.4	<0.001
**2–4**	9.7	5.3	10.0	16.6	
**Treatment (%)**					<0.001
**OP upfront**	63.6	57.6	65.7	68.3	
**Neoadjuvant CX + OP**	7.9	12.7	7.2	1.3	
**Neoadjuvant CX + no OP**	8.4	12.0	8.3	2.0	
**Palliative Cx**	20.1	17.7	18.8	28.3	
**Adjuvant CX ‡**	46.6	57.5	47.6	24.3	<0.001
**Location (%)**					0.058
***Head***	78.5	76.5	78.7	81.8	
**Corpus/tail**	21.5	23.5	21.3	18.2	
**pT ‡ (%)**					0.030
***T*0**	0.7	1.4	0.5	0.0	
***T*1**	9.9	11.9	9.0	8.9	
***T*2**	48.2	44.8	48.8	52.3	
***T*3**	38.4	38.3	38.9	36.9	
***T*4**	2.9	3.6	2.7	1.9	
**pN ‡ (%)**					
***N*0**	31.8	31.9	30.1	36.5	0.074

* As the TNM classification has been updated between 2015 and today, the N-status was dichotomized into N0 and N+. † ECOG = Eastern Cooperative Oncology Group Performance Status. ‡ only for resected cases. M = male, F = female, OP = surgery, CX = chemotherapy.

**Table 2:** Descriptive statistics for the whole cohort of octogenarians and treatment subgroups.

	Whole Cohort	Multimodal	Not Multimodal	Palliative	
	(*n* = 537, 100%)	(*n* = 95, 17.7%)	(*n* = 290, 54.0%)	(*n* = 152, 28.3%)	*p*
**Age (years, median, IQR)**	82.0 (81.0–84.0)	81.0 (80.0–83.0)	82.0 (81.0–84.0)	82.0 (81.0–84.0)	<0.001
**Sex ratio (M:F)**	1:1.2	1:1.3	1:1.4	1:1	0.310
**cT (%)**					<0.001
***Tis***	0.4	0.0	1.0	0.0	
***T*1**	9.7	17.1	12.3	2.0	
***T*2**	41.4	51.3	52.3	20.3	
***T*3**	25.7	23.7	29.5	20.9	
***T*4**	22.7	7.9	5.0	56.8	
cN * (%)					
***N*0**	60.5	62.9	61.0	58.5	0.815
**ECOG † (%)**					
**0/1**	83.4	87.4	80.0	87.5	0.069
**2–4**	16.6	12.6	20.0	12.5	
**Location (%)**					0.004
**Head**	81.8	85.3	85.2	73.0	
**Corpus/tail**	18.2	14.7	14.8	27.0	

Multimodal: surgery + neo(adjuvant) chemotherapy, not multimodal: surgery alone or neoadjuvant chemotherapy. * As the TNM classification has been updated between 2015 and today, the N-status was dichotomized into N0 and N+. † ECOG = Eastern Cooperative Oncology Group Performance Status. M = male, F = female.

## Data Availability

Data underlying this work are not publicly available.

## Supplementary Materials

The following supporting information can be downloaded at https://www.mdpi.com/article/10.3390/cancers18152423/s1, Figure S1. Overall survival stratified by age group before and after exclusion of patients with missing data on age, treatment, ECOG performance status, or survival time; Figure S2. Amount of missingness per variable in percent; Figure S3a–c. The 90-day mortality following pancreatoduodenectomy, distal pancreatectomy, or total pancreatectomy stratified by age group; Figure S4. Logistic regression with risk factors for death within the first 90 days following surgery; Figure S5. Disease-free survival after surgery of PDAC stratified by age group; Figure S6. Overall survival by treatment modality stratified by age group; Figure S7. Cox proportional hazard model for the influence of several independent variables including the received treatment on the overall survival in octogenarians; Figure S8. Binary regression analysis to identify preoperative factors associated with receiving multimodal treatment in octogenarians; Table S1. Comparison of cohort before and after exclusion of patients with missing data on age, treatment, survival data, and ECOG performance status.

## Abbreviations

The following abbreviations are used in this manuscript:

PDAC	pancreatic ductal adenocarcinoma
GCRG	German Cancer Registry Group
ADT	Association of German Tumor Centers
ECOG	Eastern Cooperative Oncology Group Performance Status
OS	overall survival
DFS	disease-free survival
PD	pancreatic head resection
DPR	distal pancreatectomy
TPE	total pancreatectomy
CI	confidence interval
OR	odds ratio
IQR	interquartile range
M	male
F	female
OP	surgery
CX	chemotherapy
