# Supplementary material for: Octogenarians and Pancreatic Cancer: Is Surgery Always the Right Choice?
Source: Cancers (Basel). 2026 Jul 28;18(15):2423. doi: 10.3390/cancers18152423 (PMC13464726; doi:10.3390/cancers18152423)
Supplement: Supplementary file 1 [file cancers-18-02423-s001.zip › cancers-4423092-supplementary.pdf]

Supplementary Figures

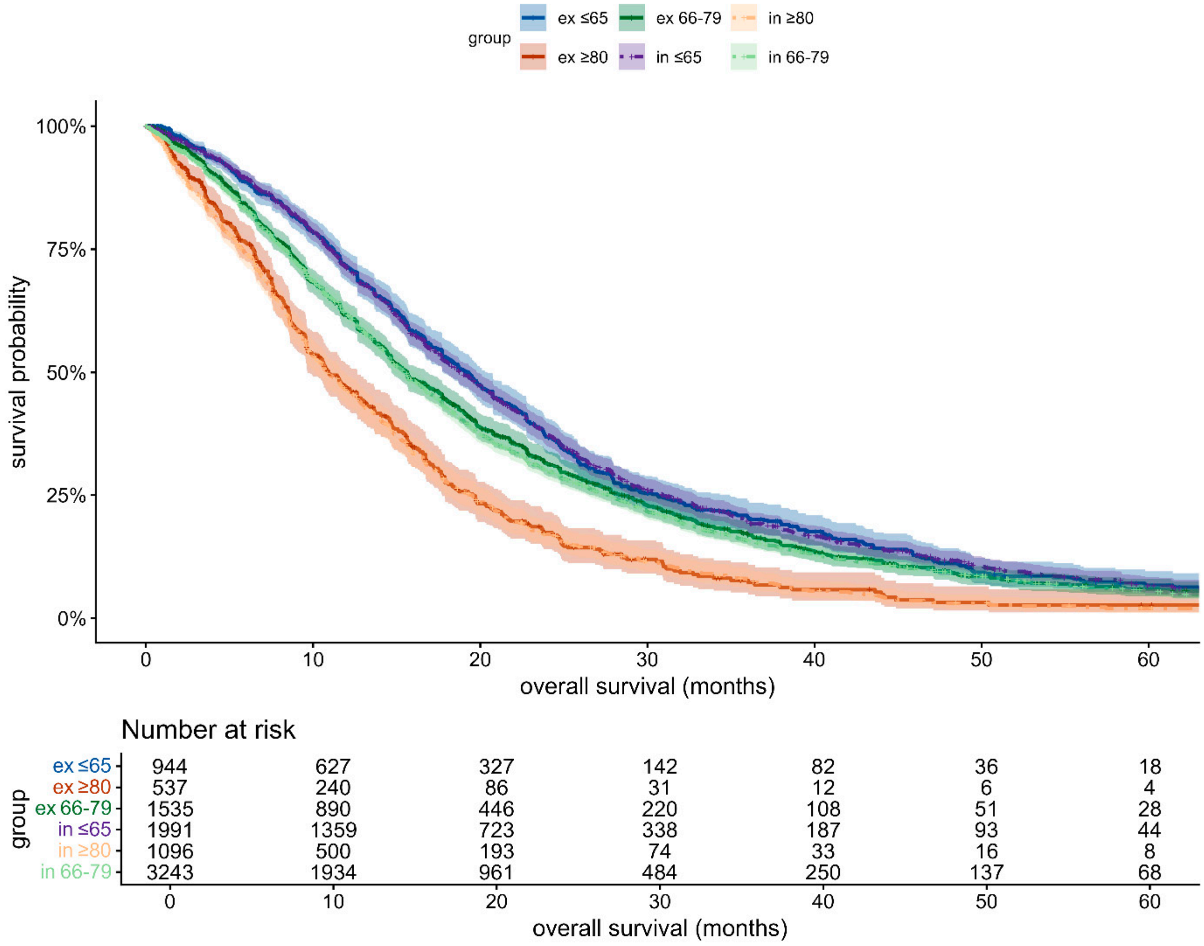

Supp. Figure S1. Overall survival stratified by age group before ("in") and after ("ex") exclusion of patients with missing data on age, treatment, ECOG performance status, or survival time.

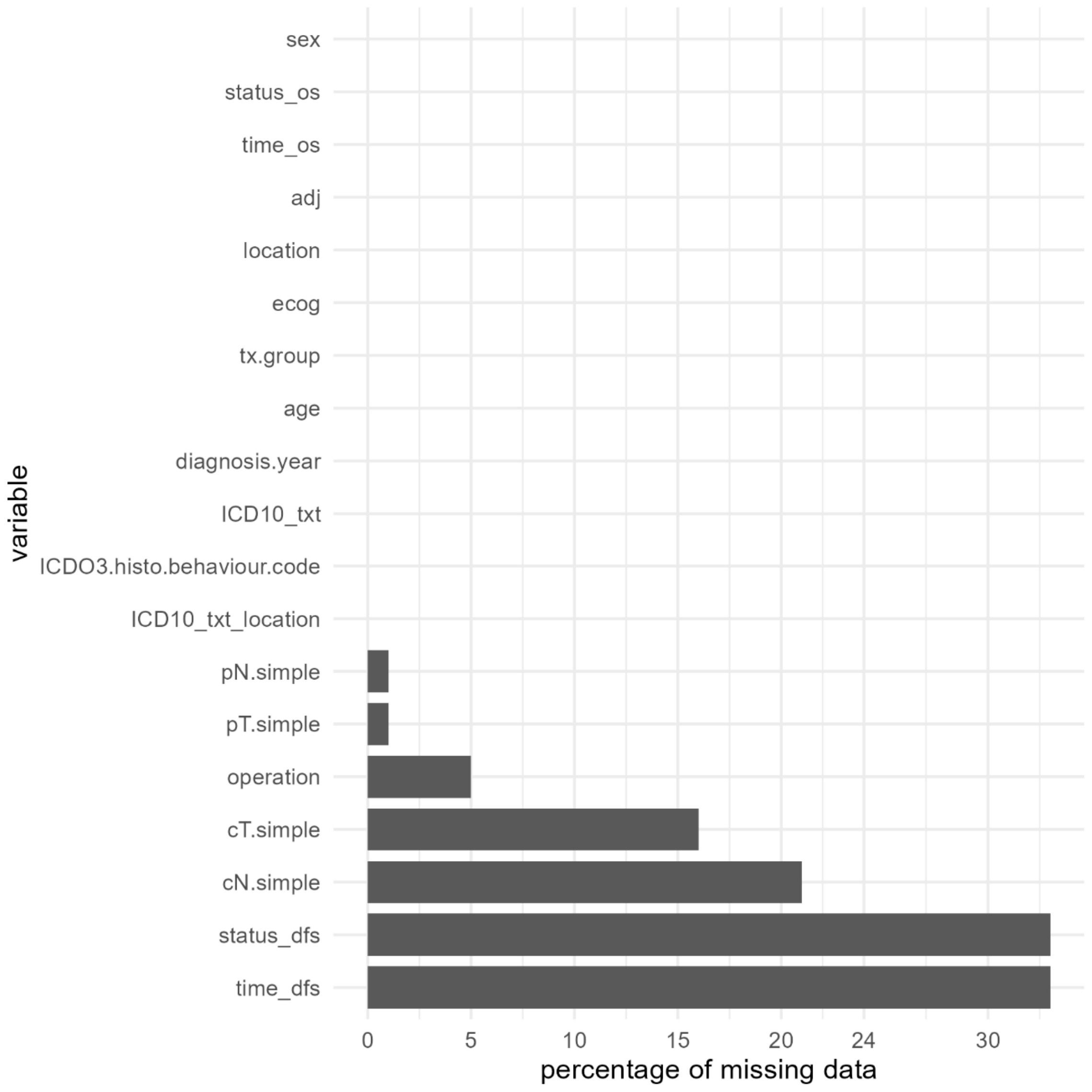

Supp. Figure S2. Amount of missingness per variable in percent. Adj = receipt of adjuvant therapy.

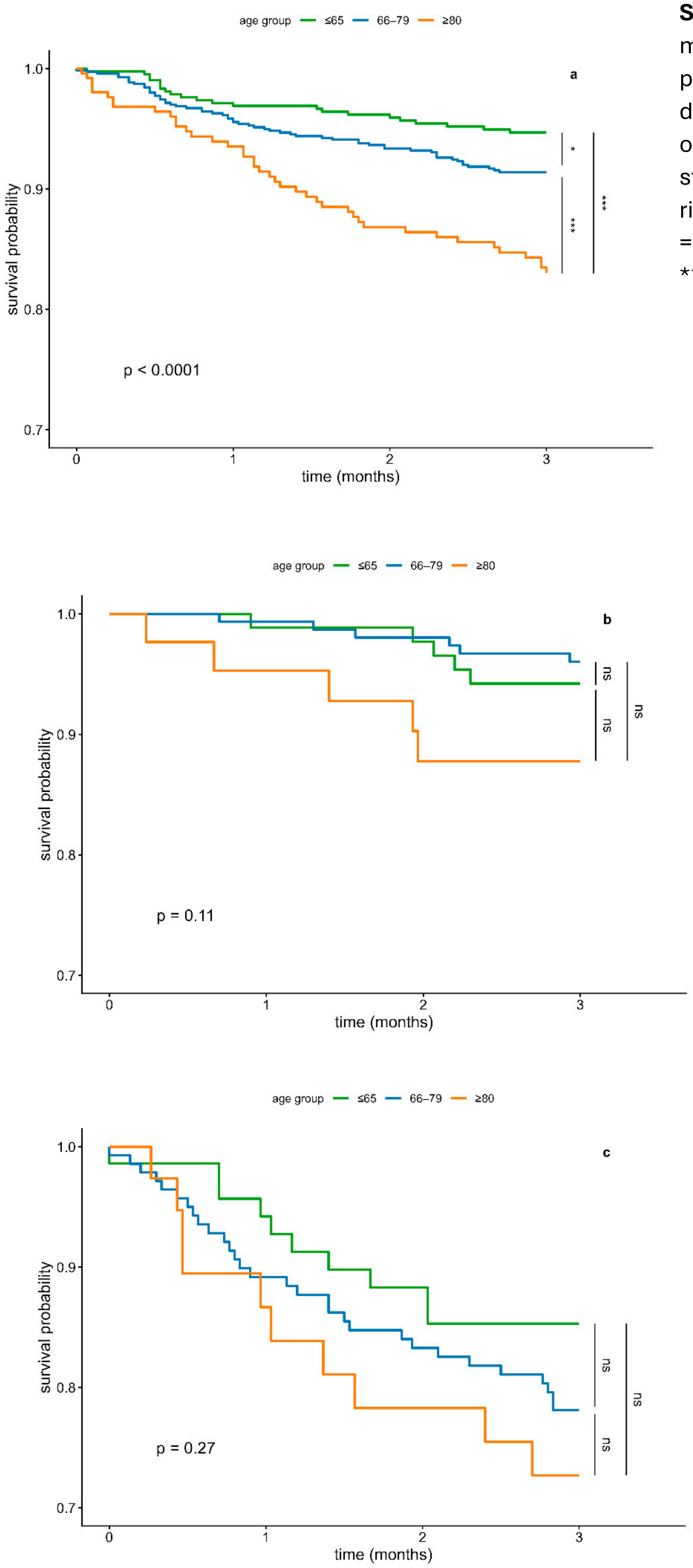

Supp. Figure S3a-c. 90-day mortality following pancreatoduodenectomy (PD) (3a), distal pancreatectomy (DPR) (3b), or total pancreatectomy (TPE) (3c) stratified by age group. Bars on the right show pairwise comparison; ns = not significant, * <0.05, **<0.01,***<0.001

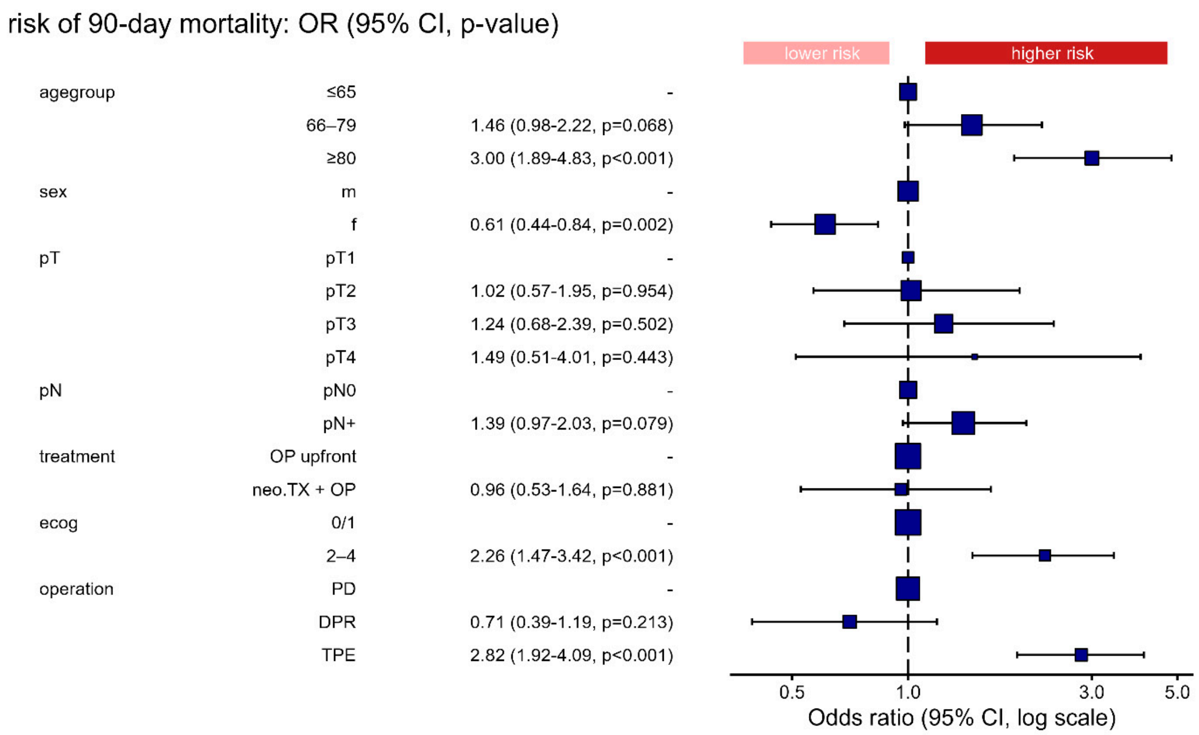

Supp. Figure S4. Logistic regression with risk factors for death within the first 90 days following surgery. Nodal status (pN) was dichotomized into pN0 and pN+. m=male, f=female, TX=treatment, OP=surgery, PD = pancreatoduodenectomy, DPR = distal pancreatectomy, TPE= total pancreatectomy

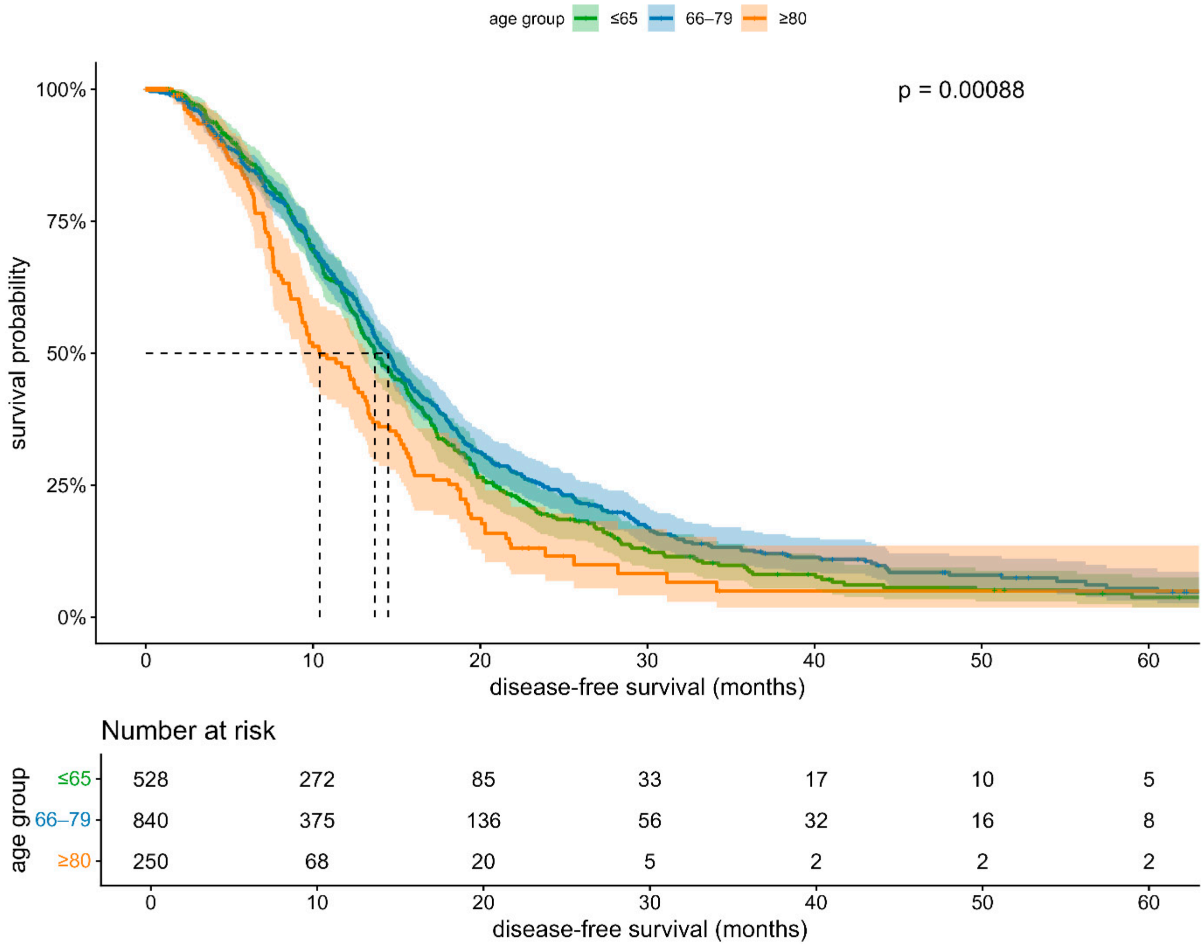

Supp. Figure S5. Disease-free survival (DFS) after surgery of PDAC stratified by age group. Median DFS ≤65 years (green) = 13.7 months, median DFS 66-79 years (blue) = 14.5 months, median DFS ≥80 years (orange) = 10.4 months, Log-rank test $p < 0.001$

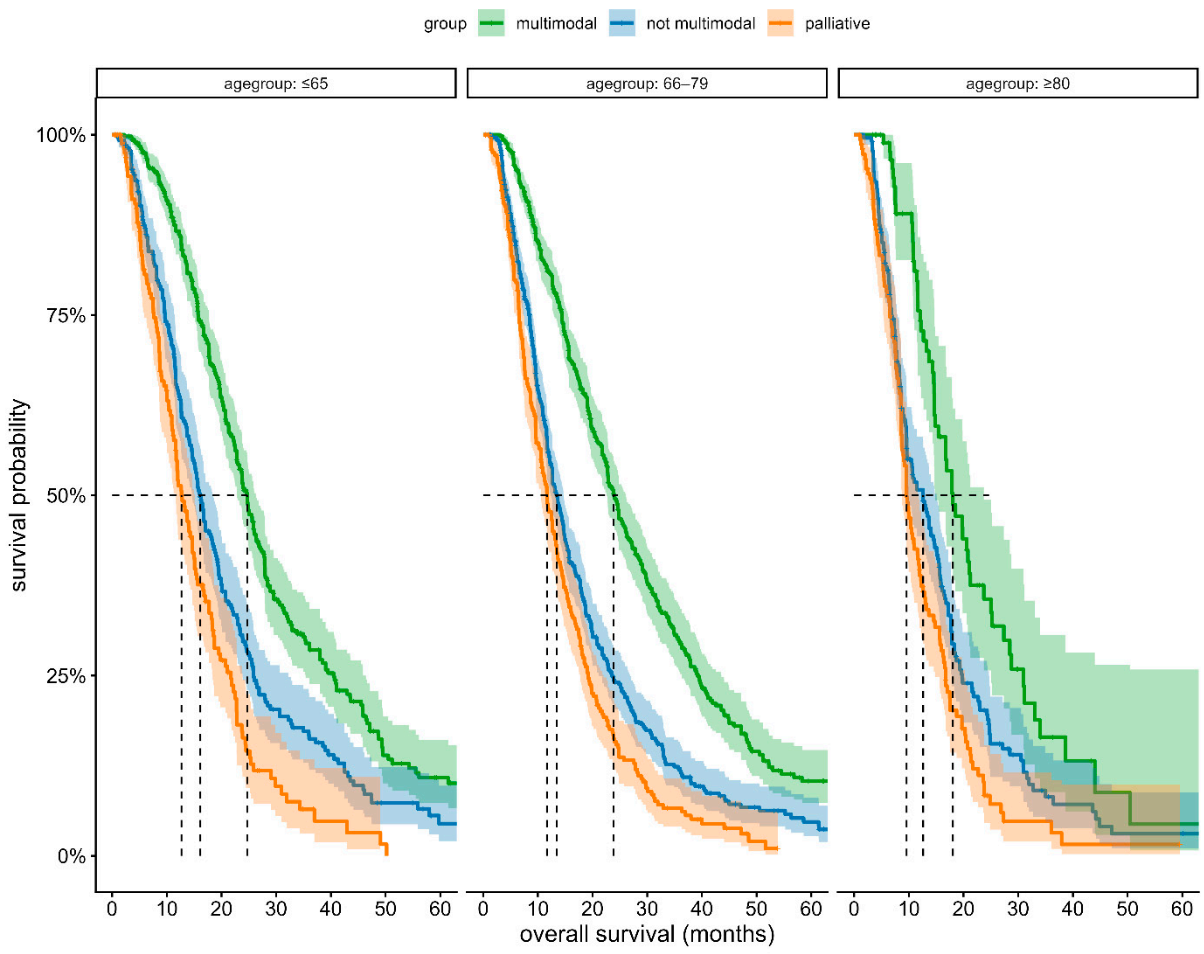

Supp. Figure S6. Overall survival (OS) by treatment modality stratified by age group.

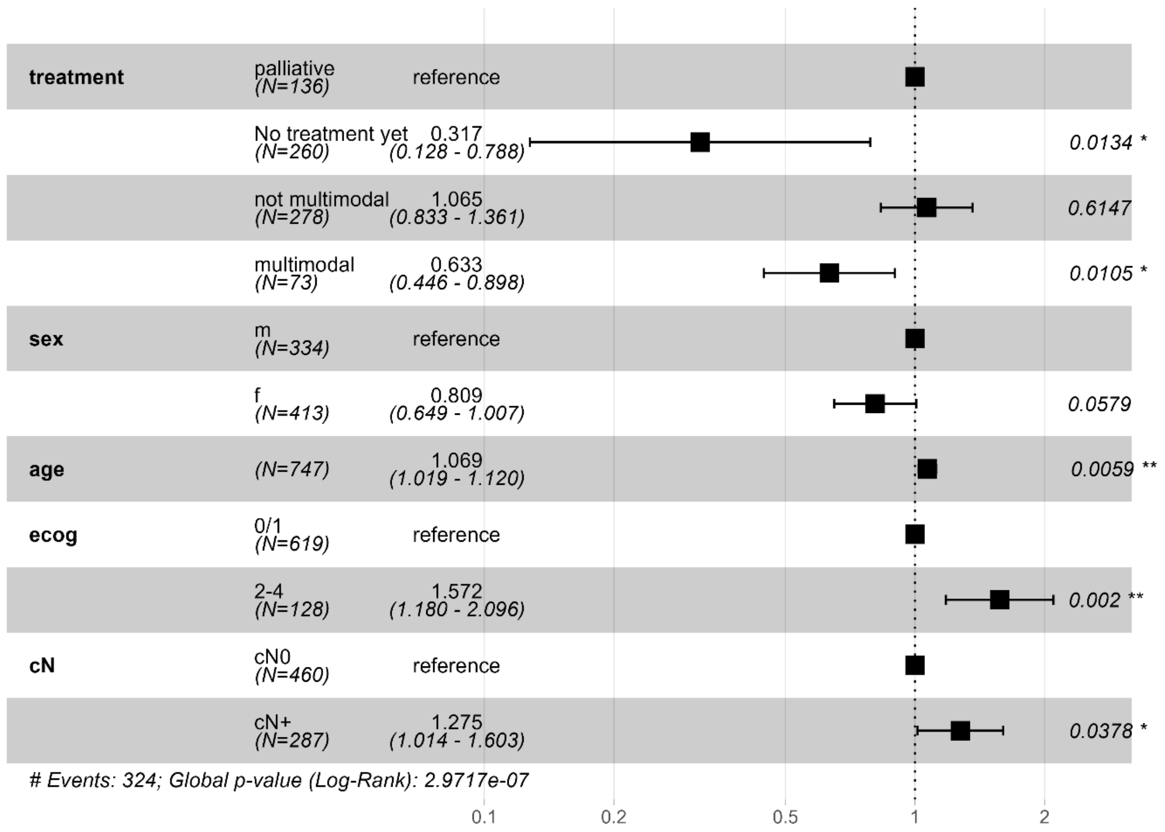

Supp. Figure S7. Cox proportional hazard model for the influence of several independent variables including the received treatment on the overall survival in octogenarians. m = male, f = female

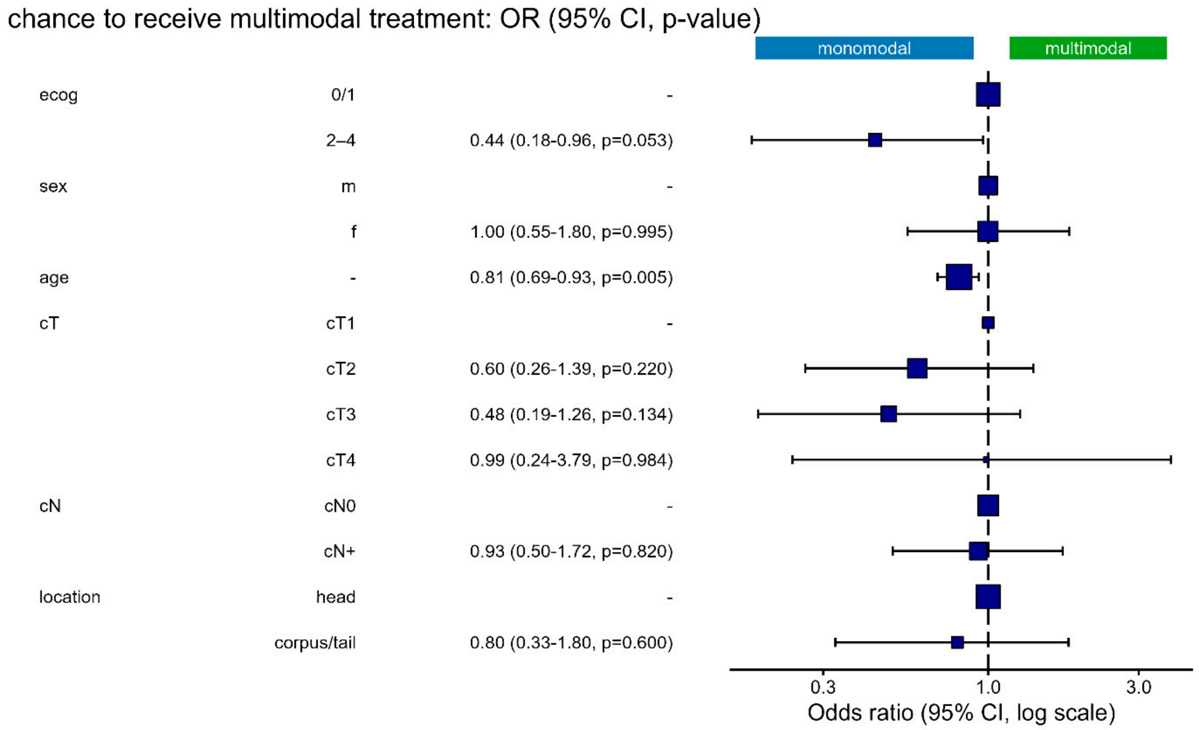

Supp. Figure S8. Binary regression analysis to identify preoperative factors associated with receiving multimodal treatment in octogenarians. Age (always above 80, as only octogenarians were included in this analysis) was included as a continuous variable. m = male, f = female

Supp. Table S1. Comparison of cohort before and after exclusion of patients with missing data on age, treatment, survival data, and ECOG performance status.

	**Before**	**After**	
	(n=6969)	(n=3016)	p
**Age (years, median, IQR)**	71 (63.0 – 78.0)	71.0 (63.0-78.0)	0.354
**Sex ratio (M:F)**	1:1	1:1	0.583
**cT (%)**			0.758
Tis	0.1	0.1	
T1	8.3	7.7	
T2	35.7	37.1	
T3	28.4	28.5	
T4	27.4	26.6	
**cN* (%)**			0.610
N0	53.4	52.7	
**ECOG† (%)**			0.662
**0/1**	90.7	90.3	
**2-4**	9.3	9.7	
**Treatment (%)**			0.007
OP upfront	67.1	63.6	
Neoadjuvant CX + OP	6.8	7.9	
Neoadjuvant CX + no OP	7.5	8.4	
Palliative Cx	18.5	20.1	
**Location (%)**			0.743
Head	78.9	78.5	
Corpus/tail	21.1	21.5	
**pT ‡ (%)**			0.885
T0	0.6	0.7	
T1	9.5	9.9	
T2	48.1	48.2	
T3	39.1	38.4	
T4	2.6	2.9	
**pN ‡ (%)**			0.973
N0	31.7	31.8	

*As the TNM classification has been updated between 2015 and today the N-status was dichotomized into N0 and N+. † ECOG = Eastern Cooperative Oncology Group Performance Status. ‡ only for resected cases. M = male, F = female, OP = surgery, CX = chemotherapy
